# Limitations of CAR-T-Cell Therapy in Hematologic Malignancies: Focusing on Antigen Escape and T-Cell Dysfunction

**DOI:** 10.3390/ijms26199669

**Published:** 2025-10-03

**Authors:** Yanyu Lin, Shuqi Luo, Jianhui Wei, Shujin Lin, Dawei Wang, Xiangqian Zhao, Zexin Feng, Yangkun Shen, Qi Chen

**Affiliations:** 1 Fujian Key Laboratory of Innate Immune Biology, Biomedical Research Center of South China, College of Life Science, Qishan Campus, Fujian Normal University, Fuzhou 350117, China; yanyulin_fjnu@yeah.net (Y.L.); qsx20231425@student.fjnu.edu.cn (S.L.); QBS20170107@yjs.fjnu.edu.cn (J.W.); wangdawei617@163.com (D.W.); zxqfjnu@163.com (X.Z.); fzx304982930@163.com (Z.F.); 2 State Key Laboratory of Cellular Stress Biology, School of Life Sciences, Faculty of Medicine and Life Sciences, Xiamen University, Xiamen 361102, China; linshujin@stu.xmu.edu.cn

**Keywords:** CAR-T, hematologic malignancies, antigen escape, T-cell dysfunction

## Abstract

Chimeric antigen receptor T (CAR-T)-cell therapy has revolutionized the treatment of hematological malignancies, yet long-term efficacy remains constrained by antigen escape and T-cell dysfunction. Recent advances have rapidly elucidated the molecular underpinnings of antigen escape mechanisms and intrinsic T-cell dysfunction, revealing novel vulnerabilities in current CAR-T paradigms. In this review, we discuss the limitations of CAR-T-cell therapy in hematological malignancies, particularly regarding antigen escape mechanisms and T-cell dysfunction. It is noteworthy that in recent years, multi-targeted CAR-T and engineered CAR-T cells have demonstrated promising clinical efficacy in overcoming drug resistance and relapse in hematological malignancies. Here, we also discuss emerging approaches to enhance the efficacy of CAR-T-cell therapy, including advanced CAR-T-cell engineering techniques, the identification of novel therapeutic targets, and the development of multi-targeted CAR-T-cell strategies.

## 1. Introduction

Currently, the primary treatment strategies for hematological malignancies include chemotherapy, radiotherapy, and hematopoietic stem cell transplantation (HSCT) [1]. Despite these developments, hematological malignancies remain a substantial global burden, contributing to a significant number of cancer-related deaths [2,3]. As knowledge regarding the molecular mechanisms of hematological malignancies unfolds, chimeric antigen receptor (CAR)-T-cell therapy has emerged as a potential game-changer, presenting innovative therapeutic options for these diseases [4,5].

CAR-T-cell therapy has demonstrated significant efficacy in relapsed/refractory (R/R) B-cell acute lymphoblastic leukemia (R/R B-ALL), non-Hodgkin lymphoma (NHL), and multiple myeloma (MM) [1,6,7,8]. Several clinical trials of CAR-T-cell therapy in R/R B-ALL patients have achieved complete remission (CR) rates ranging from 80% to 100% [9,10,11]. In 2017, the first two anti-CD19 CAR-T-cell products were approved for clinical use by the Food and Drug Administration (FDA) of the United States of America, i.e., tisagenlecleucel for pediatric acute lymphoblastic leukemia (ALL) and adult diffuse large B-cell lymphoma subtypes (DLBCL) and axicabtagene ciloleucel for DLBCL [12,13]. To date, six CAR-T products targeting CD19 or BCMA have been approved by FDA, offering unprecedented hope for these devastating diseases [1].

However, the high relapse rate of CAR-T therapy remains a crucial issue that needs to be addressed. Clinical data reveal that 29–57% of patients experience disease progression after initial response, with antigen loss and T-cell dysfunction identified as the dominant drivers of therapeutic failure [3,14]. Specifically, antigen loss accounts for 30–70% of relapses across hematologic malignancies [14,15,16,17], exemplified by CD19 downregulation/mutations in B-ALL and bi-allelic BCMA deletion in MM [18,19]. Concurrently, T-cell dysfunction manifests through exhaustion, senescence, and metabolic impairment, collectively undermining CAR-T persistence and cytotoxic function [20,21]. These processes are amplified by immunosuppressive pathways such as PD-1/PD-L1 [22,23]. Critically, the temporal dynamics governing antigen escape remain unresolved, impeding rational design of relapse-resistant CAR-T therapies.

This review looks over the current understanding of CAR-T-based immunotherapy, detailing its clinical applications across hematological malignancies, with a particular focus on the molecular mechanisms underlying therapeutic resistance issues. While early CAR-T strategies focused on single-target efficacy, overcoming relapse necessitates co-targeting antigen escape pathways and reprogramming T-cell fitness. In this context, we further explore emerging comprehensive strategies to achieve sustained disease relief.

## 2. CAR-T-Cell Therapy and the Functional Mechanism

**Figure 1:**
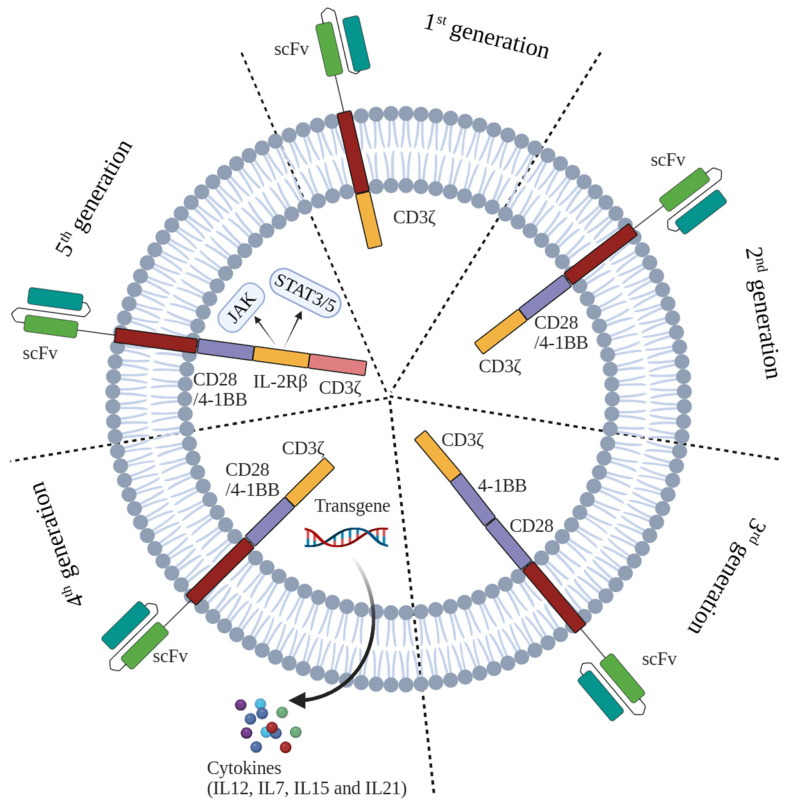
The development of five generations of CARs. The first generation of CARs contains an antigen-recognition domain scFv and an intracellular CD3ζ activation domain; it can specifically recognize tumor antigens and improve T-cell anti-tumor activities efficiently. In the second generation CARs, a costimulatory domain, such as CD28, 4-1BB, OX40, or ICOS, was integrated with the CD3ζ molecule to possess the better proliferative capacity and release more cytokines. The third-generation CAR construct encompasses two distinct costimulatory domains, such as CD28 and 4-1BB. The fourth-generation CARs are additionally modified to secrete cytokines or express suicide genes, such as IL-7, IL-12, IL-15 and IL-21; the release of these cytokines activates the endogenous immune responses in tumors. The fifth generation CARs added intracellular domains of IL-2Rβ and a STAT3-binding moiety; the activated JAK–STAT that derives from IL-2Rβ can incorporate between CD28/4-1BB and CD3ζ and improved proliferation and activation of CAR-T cells.

The concept of CAR-T-cell technology was first introduced by Eshhar et al. in 1989, suggesting the incorporation of CARs into T cells for targeted tumor antigen attacks [24]. CAR-T cells function via engineered CARs, exhibiting antigen specificity along with T-cell cytotoxicity [12,25]. A typical CAR is constructed with a single-chain variable fragment (scFv) linked via a flexible hinge domain, a transmembrane domain, and a CD3ζ activation domain [26]. Multiple generations of CAR-T-cell therapies have been engineered (Figure 1) [27]. CAR-T cells are genetically engineered T cells derived from either autologous (patient-derived) or universal (donor-derived) sources [26]. These engineered T cells express synthetic surface receptors that enable antigen-specific recognition and elimination of cancer cells, thereby initiating an antitumor immune response [28,29].

**Figure 2:**
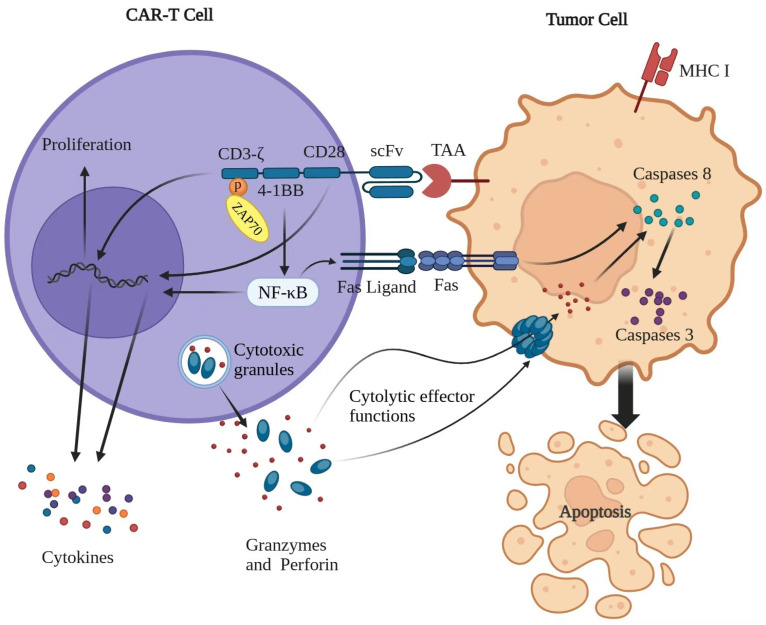
Mechanism of a CAR-T-cell killing a tumor cell. T cells mediate cytolytic effector functions predominantly by either exocytosis of cytotoxic granules containing perforin and granzymes or apoptosis induced by the engagement with tumor necrosis factor (TNF) family ligands and their respective receptor, such as Fas/FasL. Typically, tumor antigens are categorized into tumor-associated antigens (TAAs); once TAAs are identified by scFv, CAR-T cells are activated and transmit activation signals to the intracellular domain. The CD28 co-stimulatory region can rapidly activate CAR-T cells, which resulted in rapidly elevating cytokine levels and may lead to early-onset CRS. 4-1BB CARs can activate non-canonical nuclear factor-κB (NF-κB) signaling after ligand engagement, thus affecting the viability, expansion, and cytotoxicity. Moreover, 4-1BB signaling directly upregulate Fas/FasL-dependent apoptosis of T cells via NF-κB. Phosphorylation of the ITAMs in CD3ζ will initiate signal transduction via the tyrosine kinase ζ-related protein of 70 kDa (ZAP70), resulting in activated T-cell responses, such as proliferation and cytokines release. Secretion of granzymes and perforins by CAR-T cells can inhibit tumor progression. Perforin induces pore formation on the target cell membrane, facilitating the access of pro-apoptotic granzymes; granzymes can induce caspase dependent and independent apoptosis. Meanwhile, cancer-cell apoptosis will be induced by activating the apoptosis signaling pathway, including activation of the FAS-associated death domain protein. The Fas/FasL pathway results in the activation of caspase 8; the activated caspase 8 then is responsible for the processing and maturation of caspase 3, which further mediates the apoptosis program.

Upon recognition of tumor-associated antigens (TAAs) by scFv, CAR-T cells are activated, transmitting activation signals to the intracellular domain [1]. This mechanism recapitulates antigen–antibody complementarity, enabling TAAs to directly activate T cells, bypassing the major histocompatibility complex (MHC) antigen presentation [30]. CAR-T cells mediate cytolytic effector functions primarily through two mechanisms: exocytosis of cytotoxic granules containing perforin and granzymes or apoptosis induction triggered by interactions between tumor necrosis factor (TNF) family ligands and their respective receptors, such as FasL and Fas (Figure 2) [31]. The Fas and FasL pathway initiates caspase 8 activation; the activated caspase 8 is responsible for the processing and maturation of caspase 3 and further mediates apoptosis via the subsequent cleavage of more than 500 substrates [32,33,34]. A large-scale study utilizing CRISPR screening has identified signaling through FADD and TNFRSF10B (TRAIL-R2) death receptors as critical mediators of CAR-T-cell cytotoxicity, highlighting the significance of the Fas and FasL axis in CAR-T-cell-mediated tumor cells lysis.

Theoretically, perforin induces the formation of pores on the target cell membrane to facilitate the access of pro-apoptotic granzymes. Granzymes can induce caspase-dependent and independent apoptosis [35]. Granzyme B is the principal component of CTL/NK cell granules and one of the most potent among human granzymes [36]. The CTLs from granzyme-B-deficient mice demonstrate delayed target cell apoptosis kinetics, establishing granzyme B as an essential mediator of rapid and effective apoptosis of target cells. Alternatively, granzyme B mediates caspase-independent cell death by triggering mitochondrial membrane permeability [37].

In fact, CAR-T-cell-mediated cytotoxicity extends beyond apoptosis induction in tumor cells to pyroptosis, and the mechanism of CAR-T-mediated elimination diverges from that of natural T cells [38]. In contrast, the natural T-cell-mediated killing of tumor cells does not induce swelling but rather results in shrinkage, typically indicative of apoptosis. Thus, CAR-T-derived perforin/granzyme B activates the caspase 3–GSDME pathway in B leukemia cells, causing pyroptosis [38]. On the other hand, pyroptosis is key to CAR-T-therapy-induced cytokine release syndrome (CRS). Tumor cell pyroptosis triggers massive intracellular ATP release, which strongly activates macrophages to release large amounts of cytokines, leading to an inflammatory storm, which can be effectively attenuated by targeting multiple nodes of the pyroptosis. This fundamental mechanism underpins the remarkable efficacy of CAR-T cells. The following section will explore how this technology is applied across the spectrum of hematologic malignancies by targeting lineage-specific antigens, leading to the clinical successes that have revolutionized cancer therapy.

## 3. Targets for CAR-T-Cell Therapy in Hematologic Malignancies

**Figure 3:**
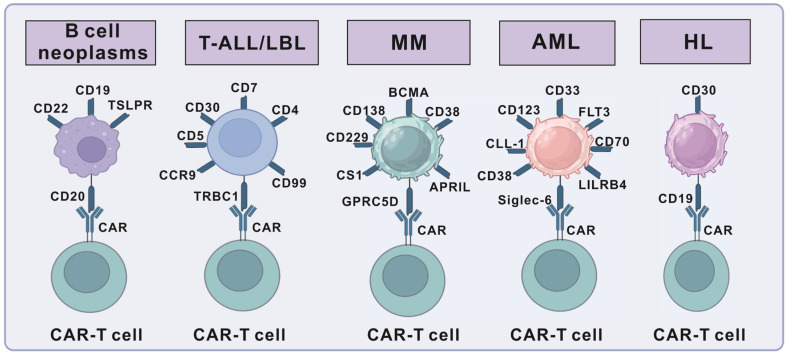
The targets for hematological malignancies. The targets for B-cell leukemia/lymphoma include CD19, CD20, CD22, etc.; for T-cell leukemia/lymphoma, the targets include CD7, CD4, CD30, etc.; for multiple myeloma (MM), the targets include BCMA, CD38, CD138, etc.; for acute myeloid leukemia (AML), the targets include CD33, CD123, CLL-1, etc.; and for Hodgkin lymphoma (HL), the targets include CD19, CD30, etc.

Hematological malignancies constitute a heterogeneous group of blood neoplasias commonly characterized by the over-proliferation of blasts in the blood, bone marrow, and other tissues, which can hinder the hematopoietic pathways [39]. Hematologic malignancies are comprehensively classified into myeloid neoplasms, lymphoid neoplasms, plasma cell neoplasms, histiocytic/dendritic cell neoplasms, childhood-type neoplasms, and genetically defined tumors based on morphological, immunophenotypic, genetic/molecular, and clinical features. The most common types include B-cell neoplasms, T-cell lymphoblastic leukemia/lymphoma (T-ALL/LBL), acute myeloid leukemia (AML), multiple myeloma (MM), and Hodgkin lymphoma (HL). Currently, the principal approved targets for CAR-T-cell therapy are BCMA for multiple myeloma (MM) [40] and CD19 for various lymphoid malignancies, including B-ALL and diffuse large B-cell lymphoma (DLBCL) [32,33]. Despite significant successes, obstacles persist that limit the expansion of CAR-T-cell therapy to other types of B-cell/plasma cell-driven hematological malignancies. Current targets under development for hematological malignancies and their potential applications are presented (Figure 3) [34].

### 3.1. Targets for B-Cell Neoplasms

The most celebrated successes of CAR-T therapy have been achieved in B-cell malignancies, leveraging targets that are highly expressed on B-cell-derived tumors while having acceptable on-target/off-tumor toxicity profiles. B-cell neoplasms represent a heterogeneous group of malignancies that arise at different stages of B-cell differentiation, ranging from precursor B-lymphoblastic leukemia/lymphoma (B-ALL/LBL) to mature B-cell neoplasms, such as chronic lymphocytic leukemia/small lymphocytic lymphoma (CLL/SLL), mantle cell lymphoma (MCL), and marginal zone lymphoma (MZL), as well as various subtypes of non-Hodgkin lymphoma (NHL). Commonly employed targets for B-cell malignancies include CD19, CD20, and CD22 [41,42]. Among these, CD19 remains the most frequently targeted and is highly expressed in most B-cell malignancies [43]. CD19-targeted CAR-T-cell therapy demonstrates remarkable clinical efficacy in relapsed or refractory (R/R) B-cell malignancies, including ALL, NHL, and CLL [44]. CD20 is overexpressed in more than 90% of B-cell lymphomas, rendering it a promising target for CD20-positive B-cell lymphomas [45]. In a phase I/II trial, anti-CD20 CAR-T cells induced CR in 54.5% of 17 patients with relapsed/refractory NHL, with a median follow-up of 20 months [46]. CD22, expressed in 90% of B-ALL cases with restricted expression in hematopoietic stem cells and normal B cells in DLBCL, is an ideal target for treating relapsed/refractory B-ALL and diffuse large B-cell lymphoma (DLBCL) [47,48]. A phase I trial of CD22-targeted CAR-T therapy achieved CR in 73% (11/15) of patients, including CD19-immunotherapy failures and those with CD19^dim^ or CD19^-^ B-ALL subtype [49].

### 3.2. Targets for T-Cell Lymphoblastic Leukemia/Lymphoma

T-cell lymphoblastic leukemia/lymphoma (T-ALL/LBL) is a single disease entity derived from transformed immature precursor T cells, presenting either as leukemia with bone marrow and peripheral blood involvement (T-ALL) or as lymphoma with predominant mediastinal and nodal masses (T-LBL), frequently with possible extension to the central nervous system [50]. The current survival rates of T-cell lymphoblastic leukemia/lymphoma patients are around 80%, but relapsed patients exhibit dismal outcomes due to acquired therapy resistance [50,51]. Compared with the outstanding clinical outcomes of anti-CD19 CAR-T-cell therapy in B-cell malignancies, the efficacy and safety of CAR-T-cell therapy in T-cell malignancies remain largely exploratory and under investigation [1].

Preclinical studies have evidenced promising activity against T-cell malignancies using anti-CD3, anti-CD4, anti-CD5, and anti-CD7 CAR-T cells [52]. Among these targets, CD5 (expressed in ~85% of T-cell malignancies such as T-LBL and PTCL) was proven by a clinical study to be effective for malignant T-cell elimination via anti-CD5 CAR-T and CD7 (highly expressed in 95% of T-ALL patients) helped most R/R T-ALL patients achieve CRs in a phase I trial of universal anti-CD7 CAR-T [53,54,55]. However, these targets (CD3/CD4/CD5/CD7) are also expressed in normal T cells, posing a risk of normal T-cell depletion [56], and additional targets like TRBC1, CD99, and CCR9 are currently being explored for CAR-T therapy in T-cell malignancies [56,57,58].

In contrast to B-cell malignancies, the development of CAR-T therapies for T-cell neoplasms faces a unique challenge: the shared expression of target antigens between malignant and normal T cells, which poses a risk of fratricide and severe immunodeficiency [58]. By leveraging the biological trait that CAR-NK cells do not express target antigens associated with T-cell neoplasms (e.g., CD7, CD5), these cells can selectively eliminate malignant T cells while avoiding both fratricide driven by shared antigens and severe immunodeficiency arising from normal T-cell depletion, thereby offering a safe therapeutic alternative for T-cell neoplasms [59,60].

### 3.3. Targets for Hodgkin Lymphoma (HL)

HL represents a distinct lymphoid malignancy of B-cell origin, with most cases originating from germinal center B cells [61] and is separated into two groups, classical Hodgkin lymphoma (CHL) and nodular lymphocyte predominant Hodgkin lymphoma (NLPHL) [62]. HL demonstrates the highest cure rates among lymphoma, with the combination of chemo-/radio-therapy, achieving 80–90% long-term remission [63]. Despite the high cure rate with initial therapy, 10–30% of patients experience relapse following initial CR [64].

HL presents B-cell-specific antigens lost and upregulation of CD30, establishing CD30 as an appropriate target for CAR-T therapy in HL patients [65]. CD30 is a member of the tumor necrosis factor receptor family, typically expressed in a small subset of activated T and B lymphocytes and up-regulated in various lymphomas [66]. In two phase I trials, patients tolerated anti-CD30 CAR-T-cell infusion without any observed side effects or toxicities. In a trial involving seven patients, CR was achieved in 28.6% (2/7) cases, with transient partial responses in 42.9% (3/7) [67]. Another trial (*n* = 18) reported partial remission (PR) in 38.9% (7/18) and stable disease (SD) in 33.3% (6/18) [43]. Despite the expression of CD30 on activated normal T and B cells, CD30 remains a promising target for CAR-T-cell therapy in HL patients due to its outstanding clinical efficacy.

### 3.4. Targets for Acute Myeloid Leukemia (AML)

AML is an aggressive hematologic malignancy characterized by the uncontrolled proliferation of immature myeloid cells in the bone marrow. While AML represents the most common acute leukemia in adults, with incidence rising sharply with age, it is also the second most common acute leukemia in children, accounting for approximately 20% of pediatric leukemia cases [68,69,70]. The application of CAR-T therapy faces perhaps its greatest challenge in AML [71]. CD33 and CD123, highly expressed on leukemic stem cells in over 90% of AML patients, are potential therapeutic targets [72]. However, phase 1 trials on these two targets showed limited efficacy, and their expression on hematopoietic stem cells could also raise concerns about bone marrow suppression [1]. Therefore, extensive preclinical research has sought to identify a plethora of antigens as potential new targets, such as LILRB4, FLT3, Siglec-6, NKG2D, CD70, LeY, CD38, and CLL-1, among others [73,74]. While these targets demonstrate overexpression on AML cells and exhibit low or no expression on normal hematopoietic stem cells, extensive preclinical and clinical investigations are still required to confirm their safety and efficacy.

### 3.5. CAR-T-Cell Therapy in Multiple Myeloma (MM)

MM serves as a typical example illustrating both the successes and limitations of CAR-T-cell therapy. It is a B-cell-derived neoplastic malignancy in the bone marrow, characterized by plasma cell proliferation and monoclonal immunoglobulin production [75,76,77,78,79,80,81]. While therapies like chemotherapy, hematopoietic stem cell transplantation (HSCT), and immunomodulatory drugs only stabilize the disease and relieve symptoms [76,77], BCMA-targeted CAR-T-cell therapy has shown definite efficacy in relapsed/refractory (R/R) MM, leading to the FDA approval of idecabtagene violence and ciltacabtagene for R/R MM [40,76,77]. However, relapse after BCMA CAR-T therapy remains frequent, making the search for new targets critical for MM treatment [1].

Several potential antigens (e.g., CD38, CD138, CD229, SLAMF7, APRIL, and G-protein-coupled receptor class C group 5 member D (GPRC5D)) have been explored as therapeutic targets [80,81]. GPRC5D, expressed in over 50% of CD138-positive malignant plasma cells in MM patients’ bone marrow, achieved a 71% objective response rate (ORR, 12/17) in 17 MM patients (including 70% of BCMA therapy-refractory cases) in a phase I study [82]. Nevertheless, safety concerns persist due to the wide expression of these receptors. For instance, CD138 is found not only on malignant and normal plasma cells but also on epithelial cells [83]. CD38, similarly, is not only highly expressed on MM cells but also on hematopoietic and activated lymphocyte cells [84]. Despite the anti-tumor effects of anti-CD38 CAR-T cells in mouse models, they adversely affect normal hematopoietic cells and lymphocytes [85].

## 4. Limitations of CAR-T-Cell Therapy in Hematologic Tumor Treatment

Despite the tremendous success achieved by CAR-T therapy, persistent challenges hinder its broader application. Several factors contribute to the failure of CAR-T-cell treatment, such as baseline characteristics (tumor bulk and burden, rapid disease progression), limitations in CAR-T-cell production (insufficient T-cell harvest, manufacturing delays, low CAR-T-cell yields), tumor heterogeneity, T-cell dysfunction (T cell-exhaustion, senescence, metabolic abnormalities, etc.), bridging therapies and lymphodepletion procedures, immune suppression within the tumor microenvironment (TME), and antigen escape. Here, we focus on the failure of CAR-T therapy due to antigen loss and T-cell dysfunction.

### 4.1. Antigen Escape

**Figure 4:**
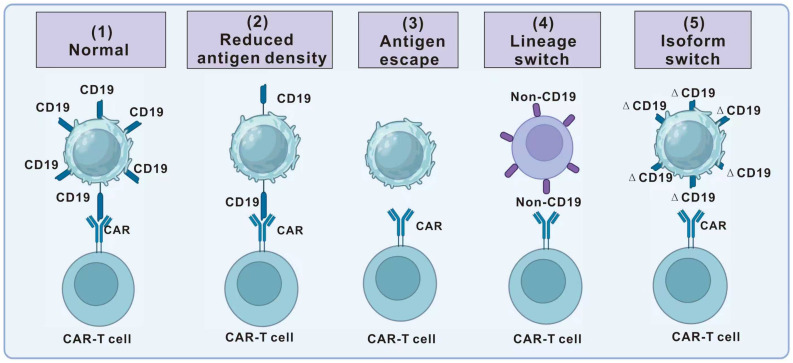
Mechanisms of antigen escape. (**1**) Under normal conditions, CAR-T cells recognize CD19 on tumor cells and eliminate them. However, the mechanisms of antigen escape in relapsed patients are as follows: (**2**) In some relapsed patients, a decrease in antigen density results in a threshold insufficient for CAR-T-cell killing, leading to tumor escape. (**3**) In some patients, a genetic mutation causes complete loss of CD19 expression, resulting in CAR-T treatment failure. (**4**) Some patients undergo tumor lineage changes where the original target disappears, leading to CAR-T treatment failure. (**5**) In some patients, a genetic mutation in the CD19 gene of the tumor cells leads to the synthesis of a truncated CD19, resulting in tumor escape.

Antigen escape has emerged as one of the most critical drivers of relapse after CAR-T-cell therapy. Although CAR-T cells can achieve high initial remission rates, 30–70% of patients across different hematologic malignancies eventually relapse due to loss or alteration of target antigens [18,86,87]. Mechanistically, antigen escape can be broadly categorized into four interrelated processes: reduced antigen density, genetic loss or mutation, isoform switching, and lineage switching, with additional contributions from reversible mechanisms such as trogocytosis (Figure 4).

Partial or complete antigen escape is a significant cause of relapse in CAR-T-cell therapy. For example, in CD22-targeted CAR-T therapy, several patients developed relapse characterized by CD22-low blasts despite persistent CAR-T cells in the bone marrow [49]. Similarly, modulation of surface antigen expression through post-transcriptional regulation has been linked to reduced therapeutic efficacy. These findings suggest that antigen density, rather than absolute presence or absence, is often a key determinant of CAR-T activity [88,89,90]. Genetic alterations represent a major mechanism of antigen escape. Complete loss of the targeted epitope frequently results from genetic alterations [91]. In B-ALL, mutations in CD19 exons 2–5 eliminate surface expression while sparing other B-cell-related genes [92]. Additionally, in multiple myeloma, bi-allelic deletion of BCMA has been observed at relapse, representing a parallel mechanism of immune escape. Together, these data indicate that irreversible genetic alterations can abolish antigen recognition and drive therapy resistance [93].

Alternative-splicing-induced target isoform switching can also lead to resistance to CAR-T therapy. Studies have identified CD19 splicing variants in B-ALL patients who relapsed after CAR-T therapy, including variants with exon 2, exon 5, or exon 6 deletions [88]. Exons 5 and 6 encode the ectodomain of CD19, which lacks the transmembrane structural domain of CD19, resulting in the loss of surface expression. Even though exon 2 does not encode the extracellular region of CD19, its deletion can also lead to therapeutic relapse. However, another study involving 12 clinical trials of CD19-negative relapses showed that the frequency of alternative splicing was extremely low (0–2.7%) when screening for relapse. Similar low-frequency was also observed in other genes, suggesting that alternative splicing is a coincidental feature for CD19 protein loss [89].

Lineage switching is another mechanism for CD19 loss in clinical trials. In rare cases, CAR-T can drive the differentiation of leukemic progenitor cells [94]. For example, B-ALL with MLL rearrangements has been reported to switch into AML following CD19-CAR-T therapy [94,95,96]. Additionally, the plasticity of pre-B cells and pre-ALL has been widely reported, such as the loss of PAX5, which has been shown to reprogram B cells into macrophages or functional T cells [97,98]. Similarly, after EBF1 deletion, transplanted primary B cells can differentiate into T cells in immunodeficient mice [99]. Similar to models with PAX5 or EBF1 deletion, the persistent use of CD19-CAR-T cells can lead to a reappearance of lineage switching in mice models that simulate human leukemia when leukemia-associated transgenes (such as ETV6: RUNX1, E2a: HLF, or MLL: AF4) are introduced into mouse hematopoietic cells to generate T or myeloid leukemia phenotypes [95]. This reflects the inherent plasticity of leukemic progenitors and represents a profound challenge for antigen-specific therapies.

Other factors can also affect antigen loss. For example, CARs induce reversible antigen loss via trogocytosis, an active process in which the target antigen is transferred to T cells, thereby reducing the target density on tumor cells and reducing T-cell activity by promoting fratricidal T-cell killing and exhaustion [100]. Additionally, disruption of CD58 in tumor cells induces the formation of suboptimal immune synapses (IS), thereby impairing the function of CAR-T cells [101]. Such dynamic processes suggest that antigen escape may not always be permanent, but can still significantly undermine therapeutic persistence.

Therefore, antigen escape is determined by multiple factors, and understanding these dynamic features provides a foundation for rational CAR-T-cell administration and combination. Given a significant proportion of patients with epitope loss escape mutations, we should consider adopting dual-targeted or multi-targeted approaches to prevent resistance.

### 4.2. T-Cell Dysfunction

**Figure 5:**
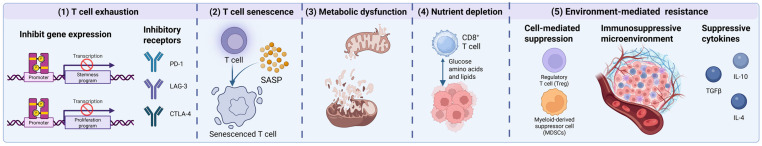
Mechanisms of CAR-T-cell dysfunction in the TME. (**1**) T-cell exhaustion: characterized by inhibited gene expression of stemness and proliferation programs and upregulated inhibitory receptors (PD-1, LAG-3, CTLA-4). (**2**) T-cell senescence: involving the generation of senescence-associated secretory phenotype (SASP). (**3**) Metabolic dysfunction: depicted by abnormal mitochondrial metabolism. (**4**) Nutrient depletion: where CD8^+^ T cells consume glucose, amino acids, and lipids. (**5**) Environment-mediated resistance: including cell-mediated suppression (regulatory T cells (Treg), myeloid-derived suppressor cells (MDSCs)), immunosuppressive microenvironment(brown represents blood vessels; blue indicates effector T-cells, such as CD8^+^ cytotoxic T-cells or CD4^+^ helper T-cells; purple denotes Tregs; light pink corresponds to TAMs), and suppressive cytokines (IL-10, TGFβ, IL-4).

The therapeutic potential of T cells primarily stems from their rapid proliferation, secretion of effector cytokines, and potent cytotoxic capabilities. However, current CAR-T clinical treatments have revealed a gradual decline and even loss of T-cell functionality. This phenomenon is mainly driven by T-cell dysfunction, which includes T-cell exhaustion, T-cell senescence, nutrient depletion (such as tumor cell consumption of glucose and amino acids), metabolic dysfunctions (e.g., impaired mitochondrial fitness), and immunosuppressive TME (e.g., upregulation of inhibitory receptors like PD-1 and CTLA-4, suppressive immune cells such as Tregs, and cytokine-mediated inhibition via factors like TGF-β) (Figure 5).

T-cell exhaustion is a central mechanism underlying CAR-T-cell dysfunction, driven by chronic antigen stimulation, an inhibitory microenvironment, metabolic reprogramming, and epigenetic modification. Key hallmarks include markedly diminished proliferative capacity (attributed to telomerase downregulation and p21/p16-mediated cell cycle arrest), impaired effector function (evidenced by decreased secretion of IL-2 and IFN-γ), and concerted upregulation of inhibitory receptors, such as PD-1, CTLA-4, and TIM-3 [102]. Studies demonstrate that persistent antigen exposure disrupts ADP-coupled oxidative phosphorylation, suppressing CAR-T-cell proliferation while upregulating exhaustion-associated genes [103]. Chronic antigen stimulation drives CAR-T cells to upregulate inhibitory receptors (e.g., PD-1, CTLA-4, TIM-3) and acquire senescence-like phenotypes, resulting in diminished antitumor efficacy [104]. Clinical evidence suggests that combinatorial use of immune checkpoint inhibitors (e.g., anti-PD-1, anti-TIM3 antibodies) can partially reverse CAR-T-cell exhaustion and enhance antitumor activity [92,105,106,107]. Furthermore, epigenetic reprogramming plays a pivotal role in driving T cells toward an exhausted state [108]. Compared to functional T cells, exhausted T cells harbor epigenetic signatures marked by altered DNA methylation and histone modifications. These changes sustain the expression of exhaustion-related genes (e.g., DNMT3A, SUV39H1, TOX, NR4A), perpetuating the exhausted phenotype [107,109].

T-cell senescence, distinct from T-cell exhaustion in its irreversibility, represents another major limitation to CAR-T therapeutic efficacy [110]. Senescent T cells are characterized by elevated senescence-associated β-galactosidase activity (SA-β-Gal^+^), upregulation of p16INK4a/p19ARF expression, and increased secretion of senescence-associated secretory phenotype (SASP) factors such as IL-6 and MMP9. Clinically, senescent CAR-T cells exhibit cell cycle arrest, impaired tumor infiltration, and metabolic inflexibility, yet paradoxically retain viability and metabolic activity [111]. This phenomenon is driven by multiple factors, including mitochondrial dysfunction and telomere attrition during T-cell proliferation [112]. Notably, studies in MM patients reveal an accumulation of senescent T cells with CD28^−^KLRG1^+^CD57^+^ or CD28^−^CD57^+^PD-1^+^ phenotypes. Intriguingly, these clonal T cells display telomere-independent senescence [113,114]. Mechanistically, senescence in human T cells is regulated by the metabolic sensor AMPK and the scaffold protein TAB1-activated kinase p38 [115]. Additionally, the ERK1/2 and p38 MARK signaling pathways cooperate with STAT1 and STAT3 to drive Treg-induced effector-T-cell senescence [116]. Accumulation of p15 and p16 proteins further accelerates this process [117]. Emerging strategies to counteract senescence include enforced expression of transcription factors such as PGC-1α or BATF, which have demonstrated efficacy in mitigating T-cell exhaustion [118,119].

During immune responses, T cells undergo metabolic reprogramming and adaptation to support their functional demands. Naïve T cells, existing in a quiescent state, primarily rely on oxidative phosphorylation (OXPHOS) for energy production. Following antigen recognition, T-cell-receptor (TCR) engagement induces a shift toward aerobic glycolysis while simultaneously enhancing OXPHOS, thereby driving the metabolic reprogramming essential for effector-T-cell differentiation. Upon antigen clearance, memory T cells reacquire a quiescent state and utilize OXPHOS to sustain their long-term persistence [120,121,122]. Studies have demonstrated that exhausted T cells exhibit impaired mitochondrial function compared to effector T cells, with diminished capacities for both glycolysis and OXPHOS [123]. This dysregulated metabolic reprogramming restricts energy supply, further compromising their proliferative potential and cytotoxic functions. Notably, augmenting mitochondrial metabolic substrates or inhibiting glycolysis significantly enhances the antitumor activity of CAR-T cells [103]. Recent research indicates that YTHDF2 promotes the progression of B-cell malignancies through a dual mechanism: by stabilizing m^5^C-modified ATP synthesis-related genes (such as ATP5PB/MG/MF) to enhance tumor cell energy metabolism, and by degrading m^6^A-modified immune-related genes (such as CD19, HLA-DMA/B) to evade immune surveillance [124]. To optimize T-cell-mediated antitumor efficacy, restoring mitochondrial fitness and functionality is critical. Targeted modulation of pathways governing mitochondrial metabolism can promote memory-like CD8^+^ T-cell differentiation, thereby improving persistence and antitumor potency [120].

In addition to these intrinsic regulatory mechanisms of T cells, the antitumor functionality of CAR-T is frequently constrained by nutrient deprivation within the TME. Tumor cells, driven by their rapid growth and proliferative rates, frequently exhibit distinct metabolic profiles characterized by heightened uptake and utilization of glucose and glutamine [125]. This altered cancer metabolism directly compromises T-cell function through competitive depletion of glucose and glutamine—critical substrates required for T-cell activation and proliferation. Furthermore, emerging evidence suggests that metabolites such as trans-vaccenic acid and mannose may enhance T-cell stemness and proliferative capacity [126,127].

The chimeric antigen receptor (CAR)-T-cell therapy targeting CD19^+^ B cells has recently been approved for the treatment of diffuse large B-cell lymphoma, showing improved results in relapsed/refractory patients. However, the initial response rate in lymphoma is much lower than that reported for leukemia [128]. This may be due to physical barriers and immune suppression within the solid lymphoma lesions. Although the tumor microenvironment is believed to play a relatively smaller role in resistance in hematological malignancies, it also contains tumor-supportive components, such as stromal cells and immune-suppressive cells [129]. To further improve response rates and ultimately prolong survival, new therapeutic approaches are needed to modulate the tumor microenvironment (TME). In fact, combination therapies with immune checkpoint inhibitors, particularly PD1/PDL1 inhibitors, have been used to improve the tumor microenvironment in hematological tumors. Results from a clinical study targeting refractory/relapsed B-cell non-Hodgkin lymphoma (B-NHL) showed an objective response rate (ORR) of 81.81% (9/11) and a complete response (CR) rate of 45.45% (5/11) with CD19 CAR-T combined with anti-PD-1 antibody (nivolumab) [130]. Furthermore, secretion of cytokines can effectively improve the tumor microenvironment, and current research is underway to integrate IL-2, IL-15, IL-7, CCL19, and IL-12 cytokine genes into CAR-T cells. Oncolytic virus therapy (OVT), as a promising non-specific tumor immunotherapy, has been clinically proven to have dual potential in specifically lysing cells and inducing anti-tumor immune responses. A study showed that oncolytic adenovirus effectively infected and lysed lymphoma, stimulating the secretion of various cytokines such as CXCL10, CCL17, CCL3, CCL4, CCL22, and CCL11, thereby enhancing the response of CAR-T cells in lymphoma [131].

Dysregulation of key signaling pathways in T cells critically impairs CAR-T-cell efficacy. Sustained activation of the PI3K–AKT–mTOR pathway has been shown to accelerate terminal differentiation and curtail the longevity of memory T cells [132]. Conversely, inhibition of mTORC1 or WNT-β-catenin signaling reduces the proportion of stem-like memory T cells (Tscm), compromising therapeutic persistence [133]. Notably, specific pathway activities exhibit profound correlations with CAR-T outcomes: the cGAS–STING axis in CD8^+^ T cells sustains stem-like properties through transcriptional regulation of TCF1, and pathway potentiation has been shown to enhance CAR-T-cell potency [134]. Paradoxically, persistent hyperactivation of endogenous STING signaling or supraphysiological STING agonist exposure impairs T-cell proliferation and effector responses [135,136]. These findings underscore the necessity for precise modulation of pathway activation dynamics and spatiotemporal control when engineering therapeutic T cells.

## 5. Therapeutic Strategies by Targeting Antigen Escape and T-Cell Dysfunction

Current strategies encompass optimization of CAR-T-cell sources (personalized and off-the-shelf allogeneic products), advanced CAR design, genetic engineering of CAR-T cells, combination therapies (with small molecules, immune checkpoint antibodies, cytokines, BiTEs, oncolytic virus, etc.), and multi-targeted CAR architectures—all of which synergistically enhance therapeutic efficacy [137,138,139,140,141]. While a substantial number of studies exist regarding source optimization and combination approaches, this review focuses on emerging solutions through genetic reprogramming of CAR-T cells to address intrinsic dysfunction, alongside multi-targeted CAR designs to counteract antigen escape or downregulation.

### 5.1. Multi-Targeted CAR-T Therapy

To prevent antigen escape, the multi-targeted combination has become the most promising strategy (Table 1). Dual-target therapy is the most frequently studied approach. Dual-targeted CAR-T-cell therapy can be achieved by generating a chimeric CAR or the sequential use of two different CAR-T-cell types.

The anti-CD19 and anti-CD20 CAR-T cells for R/R DLBCL have proven to be safe and feasible [149]. In a phase I clinical trial (NCT04007029), autologous naive T and memory T (TN/MEM) cells were engineered to express a bispecific CD19/CD20 chimeric antigen receptor (CAR; CART19/20) for relapsed/refractory NHL patients. Among 10 patients, 9 achieved objective responses [90% ORR], and 7 achieved CR [70% CR] [143]. Dual CAR-T cells targeting CD19 and CD123 have also demonstrated excellent anti-tumor efficacy and prolonged persistence, thereby preventing relapses caused by CD19 antigen loss in xenograft models [144]. Preclinical studies have demonstrated the effective treatment of AML using CD33/CD123 and CD123/CLL-1 bispecific CAR T cells [150,151]. CD19/22 dual-targeted CAR-T (AUTO3) is being used to treat relapsed/refractory large B-cell lymphoma (LBCL). AUTO3 demonstrated a favorable safety profile, with no dose-limiting toxicities or associated severe cytokine release syndrome observed. At one month post-treatment, the response rate (defined as complete remission or complete remission with incomplete hematologic recovery) was 86% (13 out of 15 patients).

Furthermore, studies have shown that sequential administration of CD19- and CD22-targeted CAR-T-cell therapy is also effective in treating B-cell malignancies [152]. In the study, 89 patients with refractory/relapsed B-cell malignancies were included, and the minimal residual disease (MRD) negativity rate was 96.0% in 51 B-ALL patients. The OR rate in 38 NHL patients was 72.2%, with a CR rate of 50.0%. Furthermore, CD19/CD37 dual CAR-T cells exhibit cytotoxicity similar to single CD19 and CD37 CAR-T cells in CD19^+^/CD37^+^ B-cell tumors but show significantly enhanced cytotoxicity against CD19 and CD37 antigen heterogeneous tumors, representing a promising treatment approach for patients with relapsed/refractory B-cell malignancies [153]. However, it is worth noting that the use of two CAR-T-cell lines may lead to an imbalance in the cell population.

In addition to the dual target approach, a three-target CAR-T approach is also under active development. A study on CD19–CD20–CD22 triple-target CAR-T cells has demonstrated their effectiveness in controlling tumors with CD19 antigen escape in vitro and a B-ALL mouse model. These CAR-T cells can be used for salvage therapy in refractory patients or as a frontline CAR-T treatment option for stubborn diseases [154].

### 5.2. Genetic Engineering to Optimize CAR-T-Cell Function

Beyond multi-targeted CAR-T development, the structural engineering of CARs to improve therapy efficacy is an inevitable strategy and has yielded transformative advancements. The therapeutic efficacy and persistence of CAR-T cells can be enhanced through precision modulation of gene expression, primarily via three strategies: modifying CAR structure, augmenting adaptive pathways, and suppressing functional inhibitory signals.

Costimulatory domain integration profoundly influences CAR-T-cell persistence and efficacy. Studies show that CAR-T cells incorporating CD27 domains demonstrate survival advantages, including enhanced effector function, proliferative capacity, and resistance to apoptosis, alongside improved persistence and antitumor efficacy in vivo [155]. MUC1D-targeted costimulatory domains enhance CAR-T durability, while ICOS intracellular domain (ICOS ICD) significantly improves in vivo persistence of CAR-expressing CD4^+^ T cells, consequently amplifying CD8^+^ T-cell longevity [156]. Comparative analyses demonstrate that CAR constructs incorporating MyD88/CD40 signaling domains surpass conventional CD28/4-1BB-based designs in proliferative capacity and antitumor potency [157,158]. Innovative DAP signaling modules (DAP10/12) further enable tailored modulation of CAR-T characteristics, including therapeutic efficacy, long-term persistence, and safety profiles [159,160,161]. Furthermore, studies have shown that CAR-T cells constructed with intracellular domains (ICD) derived from different CD3 subunits exhibit distinct tumor-killing effects. Particularly, CD3δ and CD3ε can effectively prevent CAR-T-cell exhaustion and dysfunction against different tumors [162,163]. Importantly, no universal optimal costimulatory configuration exists—ideal domain combinations require careful consideration of tumor histology, microenvironmental pressures, and patient-specific variables, as different assemblies exhibit that efficacy varies significantly in clinical scenarios.

Engineered CAR-T cells that acquire specific functions to overcome exhaustion and enhance persistence demonstrate significantly enhanced anti-tumor efficacy. Ectopic c-Jun expression renders CAR-T cells exhaustion-resistant, enhancing their proliferation and IL-2/IFNγ production, leading to superior anti-tumor activity in murine leukemia models [164,165]. Similarly, FOXO1 overexpression acts as a master transcriptional regulator, enhancing polyfunctionality, stemness, and metabolic fitness critical for durable memory-T-cell programming; while its potent anti-tumor effects are currently documented primarily in solid tumors, its potential in hematological malignancies warrants further investigation [166,167,168]. Concurrently, prolonging CAR-T-cell survival in vivo is achieved through the expression of anti-apoptotic proteins like Bcl-xL, validated in leukemia models [169,170]. In parallel, genetic manipulation introducing the CARD11–PIK3R3 fusion potently enhances cytokine release (IL-2, IFNγ, TNF) and efficacy against B-cell lymphoma [171]. Metabolic reprogramming represents another powerful axis: adenosine deaminase (ADA) promotes inosine production to enhance mitochondrial fitness and epigenetic stemness, while GLUT1 overexpression synergizes glycolytic flux with oxidative metabolism and reduces exhaustion markers [172,173,174]. Notably, GLUT1-engineered CAR-T cells exhibit efficacy not only against ALL but also suppress tumor growth in models of renal cell carcinoma (RCC) and glioblastoma (GBM). Furthermore, blocking key immunosuppressive pathways, such as TGF-β signaling via SMAD7 overexpression, has shown significant promise in improving CAR-T-cell function against solid tumors, presenting a compelling rationale for its extension to hematological malignancies [171,175].

Cytokines represent another direction for modification to enhance the function of CAR-T cells. Overexpressing cytokines (such as IL-2 signaling, inducible IL-12 armoring, and IL-23/IL-18 autocrine loops) significantly enhances CAR-T function [176,177,178,179,180,181]. This achieves localized cytokine release to reduce systemic toxicity while enhancing persistence, memory maintenance, and effector functions through autocrine signaling. Furthermore, complementary cytokine receptor engineering (e.g., IL-2Rβ signaling, CXCR2, etc.) boosts CAR-T-cell proliferative capacity and enhances tumor killing by reprogramming CAR-T chemokine-directed functions [182,183].

Another approach to enhance the efficacy of CAR-T is by knocking out inhibitory genes using TALEN and CRISPR. In 2015, a universal CD19 CAR-T product based on TALEN technology was successfully used to treat leukemia, bringing universal CAR-T technology into the public spotlight [184]. Following this, with technological advancements, CRISPR rapidly replaced TALEN to become one of the mainstream technologies in the field of gene-edited cell therapy. CRISPR-Cas9-driven ablation of immune checkpoint molecules (PD-1, LAG-3, CTLA4) alleviates T-cell activation constraints, restoring antitumor potency and prolonging persistence [185,186,187,188]. Epigenetic reprogramming via inhibition of H3K9me3 methyltransferase SUV39H1 reshapes chromatin accessibility to enforce memory differentiation trajectories. To counteract metabolic stress, GSNOR overexpression enhances mitochondrial resilience by neutralizing nitric-oxide-mediated toxicity, thereby amplifying proliferative capacity [189,190].

These strategies collectively address challenges associated with TME immunosuppression, on-target/off-tumor activity, and T-cell exhaustion, charting a course for next-generation CAR-T therapies with optimized safety and efficacy.

### 5.3. Enhancing CAR-T In Vivo Persistence and Targeting

Notably, the success of CAR-T-cell therapy in hematologic malignancies is tightly linked to the prolongation of CAR-T-cell persistence in vivo [191,192]. Sustained in vivo persistence enables CAR-T cells to continuously eliminate residual tumor cells, prevent relapse driven by minimal residual disease (MRD), and maintain long-term antitumor efficacy, making it a pivotal target for optimizing CAR-T therapy [193,194]. Although most clinical investigations focus on solid tumors, preclinical evidence strongly supports its potential application in hematologic malignancies.

CARVac represents a class of RNA-based vaccines specifically designed to amplify and sustain functional CAR-T cells in vivo [195]. In the phase 1 BNT211-01 trial evaluating CLDN6-specific CAR-T cells with CARVac in patients with relapsed or refractory CLDN6-positive solid tumors, researchers observed manageable toxicity, robust CAR-T-cell engraftment, and good tolerability of CARVac, while the objective response rate reached 57% in patients and a trend of promoting CAR-T-cell expansion was seen [193].

Targeted viral vectors have been widely used in solid tumors to overcome antigen loss—a shared challenge with hematologic malignancies [196]. For example, recombinant adenoviruses were engineered to express a CD19 tag, enabling universal labeling of antigenically diverse tumors for single anti-CD19 CAR-T recognition—achieving 100% survival and 91.78% tumor volume inhibition in premixed murine models—and enhanced efficacy by reducing established tumors and prolonging mouse survival [197]. Another engineered oncolytic viral vector was designed to tackle both antigen issues and the immunosuppressive TME. It drives ectopic expression of CD19/BCMA extracellular domains (to enable targeting by CAR-T cells) and secretes immunomodulators (CCL5, IL-12, anti-PD-1 antibody) to remodel the TME [196]. Moreover, a thymidine kinase-disrupted vaccinia virus was engineered for tumor-selective delivery of CD19, avoiding reliance on a tumor’s endogenous antigens to enable generalizable CAR-T therapy. Additionally, CD19 delivery via this vaccinia virus enhanced CAR-T activity against tumor cells with low cognate antigen expression, suggesting potential for counteracting antigen escape [194]. Unlike solid tumors, hematologic cells are more susceptible to viral transduction (e.g., B cells have high AdV receptor expression), further supporting vector-based antigen modulation as a feasible strategy to counter antigen escape in blood cancers.

## 6. Discussion

CAR-T-cell therapy achieves spectacular success against hematologic malignancies, yet long-term durability faces a critical barrier: the dual challenge of antigen escape and T-cell dysfunction. Antigen loss manifests through diverse mechanisms, including target downregulation (e.g., diminished CD19/CD22 density), lineage switch (e.g., B-ALL to AML transformation), expression of splice variants (e.g., truncated CD19 isoforms), and trogocytosis-mediated neoantigen transfer. Critically, escape variants often pre-exist therapy, evidenced by detectable CD19 mutations at initial diagnosis. Concurrently, intrinsic T-cell limitations—driven by exhaustion, senescence, and metabolic insufficiency—compromise CAR-T persistence and function. Overcoming this resilience-defining duality is therefore imperative, demanding multifaceted engineering of next-generation CAR-T constructs.

Currently, the optimization of CAR-T therapy primarily focuses on improving the co-stimulatory signaling domain of CAR structure [34,198,199]. The CD28 co-stimulatory region can swiftly activate CAR-T cells, resulting in rapid growth but hastening terminal differentiation, leading to sudden increases in cytokine levels, potentially resulting in early onset CRS [32,183,200]. In contrast, 4-1BB-based CAR-T cells exhibit slower expansion but greater persistence. 4-1BB CARs can trigger non-canonical NF-κB signaling post-ligand engagement and rely on tumor necrosis factor (TNF) receptor-associated factors (TRAFs), thus influencing viability, proliferation, and cytotoxicity [201,202]. To develop more optimal co-stimulatory domains, it is essential to conduct rigorous comparisons of various other co-stimulatory domains, including CD28, ICOS, 4-1BB, OX40, CD27, etc. [26,203]. Additionally, non-intracellular sequences can be utilized to optimize the co-stimulatory domains of CARs. Screening of a library containing approximately 2300 synthetic CARs identified a non-natural co-stimulatory domain combining TRAF and PLCγ1 signaling motifs, which enhanced CAR-T-cell cytotoxicity, persistence, and potent antitumor efficacy [204]. This research has opened up a new avenue for the modification of CAR-T cells.

Antigen escape remains a key driver of relapse following CAR-T therapy. To address the issue of antigen escape, an initial goal was to enhance the ScFv’s affinity for its target, thus improving CAR-T’s cytotoxicity against tumors. However, research shows that increasing the affinity between a CAR and an antigen does not effectively enhance CAR-T’s tumor-killing ability [110]. In contrast, enhancing antigen expression could effectively promote CAR-T’s cytotoxicity against tumors. As an example, bryostatin-1, a potent activator of protein kinase C (PKC) and an antitumor drug, could significantly increase the density of CD22 sites in precursor B-cell ALL and diffuse large B-cell lymphoma (DLBCL) cell lines, thereby enhancing the therapeutic effect of CD22 CAR-T. Another study showed that treatment with all-trans-retinoic acid (ATRA) could enhance the expression of folate receptors in tumors and enhance folate receptor-targeted CAR-T cells in treating AML [112].

While multi-target strategies offer a promising solution, defining the timing of antigen escape is critical to optimize therapeutic windows. One study analyzing relapsed patients found that genetic mutations present at relapse were undetectable in samples collected during remission, even one month prior, suggesting acquired mutations [89]. Conversely, another study detected CD19 splice variants in ALL patients at initial diagnosis, implying pre-treatment antigen loss [205]. Regardless of when escape variants emerge, the high heterogeneity of AML poses significant challenges for single-target CAR-T therapies. Encouragingly, preclinical studies demonstrate the efficacy of dual-targeting approaches and inducible CAR-T cells. Additionally, inducible CAR-T systems can mitigate toxicity associated with sustained activation while effectively eradicating tumors, potentially improving the therapeutic index for AML [206,207].

T-cell dysfunction is another cause of CAR-T therapy failure, typically associated with abnormalities in metabolism, epigenetics, and key signaling pathways. Emerging evidence supports the use of metabolic and epigenetic alterations as biomarkers to guide clinical decision-making. For instance, mitochondrial mass and membrane potential in infused CAR-T products have been shown to predict in vivo expansion and persistence. Similarly, expression of exhaustion-associated transcriptional signatures (e.g., TOX-high, NR4A-high) or DNA methylation patterns can stratify patients with higher risk of early relapse. Monitoring telomere length, NAD^+^ levels, and the prevalence of senescence-associated phenotypes (CD28^−^CD57^+^, KLRG1^+^ subsets) may further provide prognostic value. In the future, integrating these biomarkers into CAR-T product characterization and post-infusion monitoring could enable early identification of patients likely to relapse, and guide the timely application of adjunctive interventions such as checkpoint blockade, metabolic modulators, or second-line CAR-T therapy.

It is worth noting that some late-stage cancer patients do not have sufficient time to wait for the cultivation of CAR-T cells. Heavily pretreated patients often exhibit low T-cell counts, delaying or preventing leukapheresis. In a study, 22.5% (16/71) of patients had CD3^+^ T cells lower than the desired autologous lymphocyte counts for CAR-T-cell generation [208]. Some patients who are unable to receive treatment may be due to manufacturing failures, infections, deaths, or receiving alternative therapies [209]. Notably, in the BELINDA trial, response probability to tisagenlecleucel increased with CAR-T-cell dose (delivered in the dose range 0.6–6.0 × 10^8^ cells) [210,211,212].

Additionally, for patients who are unable to use or do not have a window of opportunity to use their cells for CAR-T preparation, universal cell therapy may be an optimal solution [213]. Universal CAR-T cells can be generated from a single healthy donor for multiple patients, optimized for safety and efficacy, and administered “off the shelf”, thereby offering potential cost advantages. Current research indicates that universal CAR-T therapy not only targets tumor cells but also demonstrates significant therapeutic efficacy against tumor-associated fibro-blasts [214,215]. Despite these potential advantages, the development of universal CAR-T cells lags behind autologous products due to the additional challenges of avoiding graft-versus-host disease and host-mediated graft rejection [216]. Consequently, the generation of universal CAR-T cells with reduced immunogenicity via genetic engineering or pharmacological interventions remains a pivotal research objective in this field. Additionally, since NK cells and γδ T cells inherently exhibit low immunogenicity, the development of universal CAR-NK, CAR-γδT, and CRISPR-mediated elimination of immune rejection in cell therapy offers a new direction to address this issue. Current research indicates that NK92 cell lines, umbilical cord blood, and induced pluripotent stem cells (iPSCs) can be used for CAR-NK cell production. In fact, five clinical trials have been conducted in human patients using the NK-92 cell line. In a trial using umbilical cord blood with HLA-mismatched CAR-NK therapy, none of the 11 patients experienced GVHD [59]. Therefore, for patients who are unable to use or do not have a window of opportunity to use their own cells for CAR-T preparation, universal cell therapy may be the optimal solution to address this dilemma.

In addition, determining the critical threshold of target antigen density required for optimal CAR-T-cell activation and tumor eradication is essential. Optimal CAR density achieves a critical balance between therapeutic efficacy and mitigation of off-target toxicities associated with supraphysiological dosing. Research indicates that for lytic activity, the antigen density threshold was determined to be ~200 molecules per target cell, whereas a tenfold higher antigen density, in the range of several thousand molecules per target cell, was necessary for CAR-T cells to produce cytokines [217].

Beyond antigen escape and T-cell dysfunction, manufacturing complexity and high costs are critical bottlenecks for CAR-T therapy adoption. Autologous products’ lack of scale leads to unsustainable costs, with only 10–15% of eligible patients in high-income countries accessing treatment (and <1% in low- and middle-income countries [210,218,219,220]). While universal CAR-T may reduce costs, gene editing and safety monitoring add new burdens [213,216]. Addressing these issues—via optimized manufacturing (e.g., virus-free delivery), automation, or policy interventions—is essential to make CAR-T therapy accessible and fulfill its potential for hematologic malignancy treatment [213,221,222].

In conclusion, these investigations hold significant importance in the understanding of target selection for tumor immunotherapy and the antigen escape mechanisms in recurrent patients.

## Figures and Tables

**Table 1:** Comparison of mono-target vs. bispecific CAR-T therapy in hematologic malignancies.

Target	Therapeutic Strategies	Disease	Study Phase	Response	Reference
CD19	Anti-CD19 CAR-T	R/R B-ALL	Phase 1 trial, FDA- approved	ORR 81% (61/75), CR 60% (45/75)	[18]
CD22	Anti-CD22 CAR-T	R/R B-ALL	Phase 1 trial	CR 73% (11/15)	[49]
CD38	Anti-CD38 CAR-T	R/R AML	Phase 1 trial	ORR 66.7% (4/6), CR 16.7% (1/6)	[73]
BCMA	Anti-BCMA CAR-T	R/R MM	Phase 1 trial	ORR 100% (18/18), CR 72.2% (13/18)	[79]
GPRC5D	Anti-GPRC5D CAR-T	R/R MM	Phase 1 trial	ORR 71% (12/17), CR 35% (6/17)	[82]
CD22 + CD19	Bispecific CAR-T	R/R B-ALL	IIT, Case reports	long-term remission	[142]
CD19 + CD20	Bispecific CAR-T	NHL	Phase 1 trial	ORR 90% (9/10), CR 70% (7/10)	[143]
CD19 + CD123	Combination, Bispecific CAR-T	B-ALL	Preclinical studies	Prevented antigen-loss relapses	[144]
CD19 + CD22	Bispecific CAR-T	B-ALL, LBCL	Phase 1 trial	B-ALL, ORR 100% (15/17), CR 88% (15/17); LBCL, ORR 62% (13/21), CR 29% (6/21);	[9]
BCMA + CD38	Bispecific CAR-T	MM	Phase 1 trial	ORR 87% (20/23), CR 52% (12/23)	[145]
BCMA + CD38	Bispecific CAR-T	MM	IIT	ORR 87.5% (14/16), CR 81% (13/16)	[146]
CD19 + CD20	Bispecific CAR-T	DLBCL, MCL, FL, CLL	Phase 1 trial	DLBCL, ORR 91% (10/11), CR 64% (7/11); FL, ORR 100% (1/1), CR 100% (1/1); MCL, ORR 57% (4/7), CR 57% (4/7); CLL, ORR 100% (3/3), CR 66% (2/3)	[147]
CD19 + CD22	Bispecific CAR-T	B-ALL	Phase 1 trial	B-ALL, ORR 86% (13/15)	[11]
BCMA + GPRC5D	Bispecific CAR-T	R/R MM	Phase 1 trial	ORR 86% (18/21), CR 62% (13/21)	[17]
BCMA + GPRC5D	Bispecific CAR-T	R/R MM	Phase 1 trial	ORR 100% (9/9), CR 44.4% (4/9)	[148]

## Data Availability

Not applicable.
